# Methodological issues and recommendations for systematic reviews of prognostic studies: an example from cardiovascular disease

**DOI:** 10.1186/2046-4053-3-140

**Published:** 2014-12-03

**Authors:** Janine Dretzke, Joie Ensor, Sue Bayliss, James Hodgkinson, Marie Lordkipanidzé, Richard D Riley, David Fitzmaurice, David Moore

**Affiliations:** School of Health and Population Sciences, University of Birmingham, Edgbaston, Birmingham B15 2TT UK; Department of Primary Care Clinical Sciences, University of Birmingham, Edgbaston, Birmingham B15 2TT UK; Faculté de pharmacie, Université de Montréal, C.P. 6128 succ. Centre-ville, Montreal, H3C 3 J7, QC Canada; Research Center, Montreal Heart Institute, 5000 rue Bélanger, Montreal, H1T 1C8 QC Canada; Research Institute of Primary Care and Health Sciences, Keele University, Staffordshire, ST5 5BG UK

**Keywords:** Systematic review methodology, Prognostic utility, Prognostic factor, Aspirin resistance, Cardiovascular disease, Search strategy, Study selection, Quality assessment, Reporting bias

## Abstract

**Background:**

Prognostic factors are associated with the risk of future health outcomes in individuals with a particular health condition. The prognostic ability of such factors is increasingly being assessed in both primary research and systematic reviews. Systematic review methodology in this area is continuing to evolve, reflected in variable approaches to key methodological aspects. The aim of this article was to (i) explore and compare the methodology of systematic reviews of prognostic factors undertaken for the same clinical question, (ii) to discuss implications for review findings, and (iii) to present recommendations on what might be considered to be ‘good practice’ approaches.

**Methods:**

The sample was comprised of eight systematic reviews addressing the same clinical question, namely whether ‘aspirin resistance’ (a potential prognostic factor) has prognostic utility relative to future vascular events in patients on aspirin therapy for secondary prevention. A detailed comparison of methods around study identification, study selection, quality assessment, approaches to analysis, and reporting of findings was undertaken and the implications discussed. These were summarised into key considerations that may be transferable to future systematic reviews of prognostic factors.

**Results:**

Across systematic reviews addressing the same clinical question, there were considerable differences in the numbers of studies identified and overlap between included studies, which could only partially be explained by different study eligibility criteria. Incomplete reporting and differences in terminology within primary studies hampered study identification and selection process across reviews. Quality assessment was highly variable and only one systematic review considered a checklist for studies of prognostic questions. There was inconsistency between reviews in approaches towards analysis, synthesis, addressing heterogeneity and reporting of results.

**Conclusions:**

Different methodological approaches may ultimately affect the findings and interpretation of systematic reviews of prognostic research, with implications for clinical decision-making.

**Electronic supplementary material:**

The online version of this article (doi:10.1186/2046-4053-3-140) contains supplementary material, which is available to authorized users.

## Background

Prognosis research is becoming increasingly important in health care, with a greater number of people than ever before living with chronic disease [1]. Prognostic factors relate to any measures that are associated with the risk of future health outcomes in individuals with a particular health condition [2]. Identification of prognostic factors can be potentially useful for informing a patient’s risk profile and for making therapeutic decisions [2]. The poor quality of prognostic research is however well documented, with issues relating to study design (e.g. retrospective rather than prospective), reporting (e.g. inconsistent use of nomenclature relating to prognostic research) and publication bias (e.g. preferential publication of articles with positive findings); this in turn impacts on the quality of systematic reviews and meta-analyses of published prognostic studies, which may be limited or biased in their conclusions [2, 3].

There are no accepted guidelines for undertaking systematic reviews of prognosis, and methodology is still evolving. Some methodological recommendations have been proposed by Hayden et al. [4] based on an evaluation of systematic reviews of low back pain prognosis. Recommendations include, amongst others, the assessment of all important potential biases and testing of the impact of specific biases on the review conclusions; listing studies where eligibility criteria may be unclear; and the use of sub-group and sensitivity analyses to explore sources of heterogeneity. Some recommendations for conducting systematic reviews of prognostic tests have also been made by the US Agency for Healthcare Research and Quality (AHRQ) [5] relating to, for example, defining the review question, searching, specific quality criteria to be used and extraction of summary statistics.

The Cochrane Prognosis Methods Group [6] acts as a repository for publications of prognosis methodology. It also offers advice to review authors on incorporating prognosis information into their reviews and to establish methods for undertaking systematic reviews and meta-analyses of prognosis studies; a chapter about reviews of prognosis for inclusion into the Cochrane Handbook is planned but as yet there are no specific guidelines.

Guidelines on data extraction and critical appraisal of risk prediction model studies have recently been published [7], and it is likely that some issues may overlap with prognostic studies.

This article explores the methodology of systematic reviews of prognosis in a cardiovascular area. The clinical question related to the prognostic utility of platelet function tests (PFT) for the detection of ‘aspirin resistance’ in patients with established cardiovascular or cerebrovascular disease; more specifically, whether insufficient platelet function inhibition by aspirin (‘aspirin resistance’) as defined using one of several PFTs was associated with an increased risk of adverse clinical outcomes. A further aim was to identify whether individual patients at greater risk of future adverse clinical events could be identified through PFTs. In this example, the prognostic factor (‘aspirin resistance’) is defined by the result of a clinical (diagnostic) test result (i.e. an individual is designated as ‘aspirin resistant’ or ‘aspirin sensitive’ using a PFT), so either ‘aspirin resistance’ or the PFT result could be considered to be the prognostic factor, as they are both describing a state of platelet reactivity.

A ‘systematic review of systematic reviews’ was undertaken as part of a wider project, which comprised a new systematic review on the same topic and an economic evaluation [8, 9]. Both the new systematic review and the previous systematic reviews are used as illustrative examples throughout. The aim of this article was not to critique individual reviews but to explore and compare the different methodological approaches employed within these reviews, examine whether different methodological approaches can affect overall findings and conclusions drawn, and draw out common methodological considerations which may be useful for informing future systematic reviews of prognostic factors.

## Methods

The search for systematic reviews [9] was performed in April 2012 and updated in May 2014 (see Additional file 1 for sample search strategy). Systematic reviews were eligible for inclusion if their primary aim was to examine or quantify the potential association between ‘aspirin resistance’ (a candidate prognostic factor, which is defined by a PFT) and risk of future cardiovascular events in patients who were prescribed aspirin therapy. Further inclusion criteria were as follows: reporting of search strategy and at least one other methodological component (e.g. details on study selection process or quality assessment) and all types of PFT eligible (i.e. reviews focussing on only one specific PFT were excluded). The following components of the reviews were then compared and key differences tabulated (where reporting allowed):

-  Volume of evidence
-  Search strategy
-  Eligibility criteria
-  Quality assessment strategy
-  Reporting of results.

Potential impacts on findings and implications for future systematic reviews of prognostic factors were discussed.

In a separate exercise, and in order to further explore different approaches to search strategies, different validated prognostic search filters were also added to the original search strategy of one systematic review [8] in order to assess the effect on sensitivity and precision of the search.

## Results

Eight relevant systematic reviews were identified, with publication dates from 2007 to 2014 (see Additional file 2 for review selection process and characteristics of systematic reviews): seven [10–16] through the searches and the HTA report/new systematic review [8] itself. All eight systematic reviews had the same aim, namely to explore the association between ‘aspirin resistance’ and the risk of future cardiovascular events in patients on aspirin therapy for secondary prevention.

### Volume of included studies and method of determination of prognostic factor (platelet function test)

The number of included primary studies and the consistency between the eight reviews were explored. Primary studies with a publication date up to 2006 were considered, in order to make the search periods comparable across reviews. Thirty-eight unique studies were included across these reviews for this period. The numbers of studies included in individual reviews varied between 2 and 25 for the time period up to 2006 (see Additional file 3 for number and overlap between reviews). No study was included in all eight systematic reviews. Only four studies were consistently represented in 7/8 (88%) of the systematic reviews, six studies in ≥75% of the reviews, nine studies in ≥63%, 14 studies in ≥50%, with the remainder of primary studies (*n* = 24) represented in only 1–3 of the eight reviews. The prognostic factor can be measured by a number of different methods (PFTs), and no review restricted inclusion to a specific PFT. As a consequence of including different primary studies, varying proportions of studies using a particular method (PFT) were represented in the eight reviews (Figure in Additional file 4). Different inclusion criteria unrelated to the type of PFT are thought to have contributed to these discrepancies to some extent and are further discussed below. The systematic review by Li et al. [15], for example, included only studies where compliance had been verified. However, it does not appear that differing selection criteria explain all of the considerable variation. Reliably identifying prognostic studies continues to be a problem in systematic reviews of prognostic factors.

### Search strategies

Search strategies, in particular with regard to filters for study identification, are less well developed in the prognosis field and potentially less able to identify relevant studies [17]. This appears to be reflected within this example in the number and type of search terms, as well as combinations of terms, which varied substantially between the reviews. Table 1 shows the types of search terms included in the respective search strategies. None of the reviews reported use of a filter for prognostic studies, though the HTA report [8] incorporated search terms relating to prediction and prognosis. There were similarities in terms of sources searched, with all searching at least two major electronic databases (such as MEDLINE, Embase, the Cochrane library) and using citation checking. Where studies specified the initial number of citations identified with their search strategy, the numbers varied greatly: 36,573 [11], 16,583 [8], 3,847 [15], 3,882 [16] and 1,978 [13], a reflection of the sensitivity (breadth) of the initial search. Two reviews [10, 11] attempted to reduce the number of hits by subsequently including additional limits. The initial search yield did not necessarily appear to correlate with a greater number of included studies; however, a formal assessment of this across reviews was not possible due to a lack of clarity in the reporting of yields at different stages.

**Table 1:** **Search terms used in the systematic reviews**

Search terms relating to	Systematic reviews*						
	HTA report [8]	Li et al. [15]	Pusch et al. [12]	Sofi et al. [14]	Krasopoulos et al. [11]	Wisman et al. [16]^+^	Canivano and Garcia [10]
Aspirin	✓	✓	✓	✓	✓	✓	✓
Resistance	✓	✓	✓	✓	✓	✓	✓
Platelet (function)	✓	✓	✓	✓	✓	✓	
Outcomes/condition	✓	✓	✓	✓			
Names of PFTs	✓						
Prognosis/prediction	✓						
Filter for study design							

*Search terms not listed in the systematic review by Snoep et al. [13]. ^+^Not clear if all search terms listed.

In order to test whether the introduction of a prognostic filter could have limited the sensitivity (and reduced the number of hits), whilst retaining the precision (i.e. identify relevant studies), the literature searches for the HTA report [8] were rerun, for MEDLINE only, with additional validated Haynes filters relating to prognosis and clinical prediction [17]. It was found that the ‘prognosis’ Haynes filter picked up 77% of studies identified by the original broader search strategy whilst reducing the volume of overall hits to 82%; the ‘clinical prediction guide’ Haynes filter picked up 62% of studies, whilst reducing the volume to 72%. These results show that studies meeting the eligibility criteria would have been missed using either of the prognosis/prediction filters, though they both significantly reduced the larger initial quantity of evidence. In the current context, it is unlikely that citation checking (forward and backward) would have led to the identification of the missed studies, but this remains to be empirically tested.

### Inclusion criteria

There were variations between reviews both in the level of reporting of inclusion and exclusion criteria and the actual criteria applied. Only three reviews [8, 11, 16] gave details on whether patients who were receiving aspirin as monotherapy and/or dual therapy (aspirin + other antiplatelet agent) were eligible; this distinction is important as the presence of a second antiplatelet agent may interact with the prognostic factor assessment (platelet function) and also be associated with the outcome of interest (cardiovascular events).

Further, only 5/8 [8, 11, 14–16] reviews specified whether prospective and/or retrospective study designs were eligible. Both concomitant therapy and study design are factors that may have an effect on results, and if not specified, may introduce bias into overall findings.

Further, the terms prospective or retrospective may be used to describe the study design but may not apply in the same way to data collection/analysis. For example, sample collection may have occurred before the outcome of interest, but analysis of the sample (i.e. measurement of the prognostic factor by PFT) may have been undertaken after the outcomes occurred. One such example is the study by Eikelboom et al. [18], which is described as a nested case–control study (within an RCT) but could also meet the criteria for a prospective (prognostic) study as sample collection preceded clinical events.

Other eligibility criteria variously specified in individual reviews only were the reporting of specific outcome statistics [14], blinding of investigators [11] measurement of patient compliance [15] or English language [16]. Again, these criteria may have an impact on completeness and robustness of the evidence identified and synthesised by each review.

Regardless of how eligibility criteria have been defined, screening for eligibility may be hampered by poor reporting. The HTA report [8] found that the levels of reporting within primary studies varied dramatically in terms of whether (i) the results of the PFT (for assessment of prognostic factor) were reported, (ii) results were dichotomized (‘resistant’ and ‘sensitive’), (iii) cardiovascular outcomes were reported, and (iv) outcomes were linked to ‘resistant’ and ‘sensitive’ groups. Not all of this information was necessarily discernable from the abstract, thus necessitating a detailed reading of whole articles at an early screening stage.

Differences in selection criteria can lead to different included studies, and as a result, different conclusions as to the prognostic utility of a factor of interest. Even where included, studies are consistent across reviews, the quality of reporting within included studies may also influence any conclusions drawn by review authors.

### Quality assessment of included studies

Quality assessment of primary prognostic studies is a developing field, and the different approaches used across the eight reviews may in part be a reflection of their publication date. Both quality assessment (where undertaken) and use of quality findings in interpreting results were highly variable within this example (see Table 2). Three reviews used an item related to study quality as an eligibility criterion, e.g. ‘investigators blinded to patients’ aspirin status’ [11] which is in contrast to effectiveness questions where quality assessment can, but does not usually, form part of the study selection process. This may be problematic where such quality items have not been reported in the article.

**Table 2:** **Quality assessment approaches**

Review	Quality assessment undertaken and method	Findings presented/use of summary score	Findings used in context of results/sensitivity analysis	Comment
Canivano and Gracia [10]	None	N/A	N/A	
HTA report [8]	Quality assessment tool derived from QUADAS [19] and the Hayden checklist relating to prognostic studies [20]	Results of the quality assessment were presented	Impact commented on but sensitivity analyses not deemed possible.	
Krasopoulos et al. [11]	Study eligibility criterion: investigators to be blinded to patients’ aspirin status	Quality rating for risk of bias (A to D) but not explicit on how this was derived	No	Terminology used was confusing (e.g. ‘allocation of blindness’ and ‘compliance with blindness’). The term ‘allocation concealment’ used in the context of observational studies is not appropriate
Li et al. [15]	Study eligibility criterion: only those studies with verified compliance. Newcastle-Ottawa checklist [21] for cohort studies	Findings presented	No	
Pusch et al. [12]	None	N/A	N/A	
Sofi et al. [14]	Study eligibility criterion: prospective study design	N/A	N/A	
Snoep et al. [13]	Quality criteria relating to: control for confounders, measurement of exposure, completeness of follow-up and blinding, and, for case–control studies, matching and case definition	No	No	
Wisman et al. [16]	Modified QUADAS tool [19] (for quality assessment of diagnostic accuracy studies). 11 items assessed	Findings presented	Sensitivity analysis. Studies scoring ‘low risk of bias’ on eight or more of the quality items were considered to be good quality	

Prognostic studies may be of varying study design, e.g. cohort or case–control design, or one arm of an RCT, and may include a diagnostic test (such as a PFT) for identifying prognostic factors, and this is reflected in the choice of different quality assessment tools. Two of the reviews (Wisman et al. [16] and the HTA report [8]) modified one or more checklists to include items relevant to the particular topic area. The HTA report based quality assessment on QUADAS (quality assessment of diagnostic accuracy studies) [19] for assessing quality of test accuracy studies and guidelines for appraising prognostic studies [20], the latter of which has since been further developed into the Quality in Prognosis Study (QUIPS) tool [22]. It is important that quality assessment focuses on those aspects relevant to the prognosis question rather than be (solely) led by the study design, particularly where the prognostic aspect may not have been the main or only focus of the study.

### Presentation of results

Primary studies mainly reported results as dichotomous frequency data, i.e. the number of clinical events in ‘aspirin resistant’ (prognostic factor positive) and ‘aspirin sensitive’ (prognostic factor negative) arms, where a threshold value was used to define the two groups. Fewer studies reported adjusted/unadjusted odds ratios (OR) and/or hazard ratios (HR). The extent to which data was transformed for use in meta-analysis (or presentation in forest plots) was variable across reviews. All reviews calculated RRs or ORs from frequency data. The test results for the prognostic factor were in the form of continuous data (i.e. level of platelet aggregation), but these were frequently dichotomised into positive/negative using a threshold, or presented as tertiles or quartiles. There was often no explanation regarding the choice of threshold.

The HTA report [8] presented results for multiple (PFT) thresholds for designating ‘aspirin resistance’ (presence of prognostic factor) where reported; calculated thresholds where PFT test results were reported as tertiles or quartiles by collapsing these into two groups: presented HRs where available and presented adjusted results where available. Adjusted results can reveal whether a test has prognostic utility over and above other prognostic factors. None of the included studies reported prognostic models for individual risk prediction (i.e. a model containing PFT results and other prognostic factors for absolute risk prediction), so findings were limited to an average association between the prognostic factor and outcome. Few systematic reviews considered adjusted results or the potential importance of models.

Time-to-event analyses may be more appropriate when accounting for different lengths of follow-up; HRs were presented in the HTA report [8], which also calculated HRs from other available data where possible (using methods described by Parmar et al. [23] and Perneger [24]). In contrast, two of the other reviews (Li et al. [15], Sofi et al. [14]) converted HRs to relative risks (RR) for inclusion in synthesis using meta-analysis. There is a balance between maximising data for analyses (improving effect estimates) and the assumptions that have to be made in order to do this (limiting conclusions that can be drawn). Presenting the same data using as many outcome statistics as reported or calculable does allow exploration of whether results are sensitive to the choice of outcome statistic.

Five reviews undertook meta-analyses on variously defined composite cardiovascular outcomes (major adverse cardiac events (MACE)) [15], composite cardiovascular endpoints [16], clinical ischaemic events [13] or any cardiovascular events [11, 14]) while only one [13] also presented individual outcomes (re-occlusion and myonecrosis after PCI) (fixed or random effects assumptions were used and a pooled RR or OR presented (see Table 3). All main analyses included all studies despite evidence of moderate to substantial statistical heterogeneity [25] for three (*I*^2^ values of 31% [15], 49% [13], 63% [14], 69% [11] and 77% [16]). This may be problematic when trying to draw conclusions for a particular method (PFT) for determining the prognostic factor, patient type or outcome; moreover, including large numbers of studies in a meta-analysis may lend a spurious robustness to the overall pooled estimate but do little in terms of informing clinical decisions due to large inconsistencies (heterogeneity). All five reviews considered heterogeneity using a number of different approaches; the most common was sub-grouping by PFT with some reviews also considering outcome, patient characteristics or type of antiplatelet therapy (mono or dual). One review [15] attempted to limit heterogeneity *a priori* by having more stringent inclusion criteria but did not sub-group by type of PFT. Having to deal with heterogeneity is not unique to prognostic research, but an added layer of heterogeneity may have to be considered as prognostic factors could be measured in multiple ways, using multiple thresholds, at multiple time-points and adjusted for differing sets of other prognostic factors. Using a fixed effects model may be particularly problematic for prognosis studies given the issues around heterogeneity, and its use in two reviews [11, 15] was probably not justified. There is a danger that without careful consideration of heterogeneity, pooled estimates may give an impression of precision and certainty that is not justified.

**Table 3:** **Approaches to analysis**

Results reported	Canivano and Gracia [10]	HTA report [8]	Li et al. [15]	Krasopoulos et al. [11]	Pusch et al. [12]	Snoep et al. [13]	Sofi et al. [14]	Wisman et al. [16]
Narrative/results tabulated only	✓				✓			
Tabulated by PFT					✓			
Forest plot (no pooling)		✓						
Meta-analysis (fixed effect)			✓	✓				
Meta-analysis (random effects)			✓ (As sensitivity analysis)			✓	✓	✓
Pooled RR			✓ (As sensitivity analysis)				✓	✓
Pooled OR			✓	✓		✓		
HRs reported		✓ (Not pooled)						
Meta-analysis by PFT				✓ (For some)			✓	✓
Meta-analysis by outcome				✓		✓		✓
Meta-analysis by mono- or dual therapy				✓				✓
Meta-analysis by duration of follow-up							✓	✓
Meta-analysis by aspirin dose				✓		✓	✓	✓
Meta-analysis by population							✓	✓
Sensitivity analysis (quality)								✓
Results by different thresholds presented		✓						
Adjusted/unadjusted results presented		✓						

## Discussion

Systematic reviews of potential prognostic factors are on the increase, not least due to the rising interest in personalised medicine. Some guidance on how best to conduct such reviews exist [4–6] but is still evolving, and it is apparent that recommendations have not yet been widely adopted by systematic review authors. It is hoped that considerations presented in this article will further inform and extend what could constitute ‘good practice’ in systematic reviews of prognostic factors. Key considerations are summarised in Table 4.

**Table 4:** **Considerations when undertaking systematic reviews of prognostic factors**

Considerations	Description
Primary study identification	Studies are not necessarily ‘badged’ as prognostic/predictive and a variety of terms are inconsistently used (e.g. risk, association, relationship etc.)
	Using prognostic filters substantially reduces the volume of search hits, but it is likely that relevant studies will be missed
Study selection	Selection criteria are not consistently reported. This may be particularly important in terms of specifying study design (retrospective/prospective)
Hierarchy of studies	Where large numbers of (poor quality) primary studies are identified, a step-wise approach to inclusion may be feasible: (i) inclusion only of studies reporting a prognostic model/ results adjusted for other prognostic factors, (ii) inclusion of prospective studies reporting on a single prognostic factor and (iii) inclusion of all studies reporting on a single prognostic factor
Definition of prognostic factor	If identifying a potential prognostic factor is dependent on a diagnostic test, then diagnostic accuracy aspects of one or more tests may need to be assessed in a separate exercise (the QUADAS tool [19] may be appropriate for this)
	Consider whether it is clinically appropriate to dichotomise prognostic factor or whether it should be used as a continuous variable (particularly in a model)
Quality assessment	The QUIPS tool [22] should be used to inform quality assessment rather than tools relating to specific study design; further tailoring may be necessary depending on topic specific issues
Analysis	Meta-analysis should only be undertaken after extensive consideration of clinical and methodological heterogeneity
	Data for meta-analysis can potentially be maximised by converting outcome statistics, which may also allow exploration of sensitivity of results to use of statistics
	Meta-analysis results should be made specific to particular threshold values or ideally for the factor left on its continuous scale
	Adjusted results should be presented where possible
	Time-to-event analyses should be considered when accounting for different lengths of follow-up
	Small study effects (potential publication bias) should be examined in those meta-analyses containing ten or more studies
	Models based on individual patient data should be considered

Identifying all relevant prognostic studies for inclusion into a systematic review is a time-consuming process. The search strategy, including the type of study sought, should be guided by the review question, and there may be important differences depending on whether evidence is sought on one, or several, known or potential prognostic factors, one or more outcomes associated with those factors, and whether the question is related to proof of concept, prospective clinical validation, incremental predictive value or clinical utility [26].

It is known that published prognosis search filters have lower sensitivity and precision values than filters used for effectiveness questions [27], probably a result of variable terminology used in primary studies and a lack of consistent indexing. In this example, the research question of relevant primary studies was variously described as ‘aspirin resistance associated with clinical events’ [28], ‘…to determine the event rate in aspirin responders and non-responders’ [29], ‘the role of aspirin resistance on outcome’ [30] and other variations. Certainly, it appears (from this example) that some studies are more likely to be identified than others, thus potentially weighting the evidence base in their favour, particularly where several reviews have been undertaken.

Whilst very broad search strategies are likely to identify more relevant studies, screening studies can be very time consuming. Approaches such as ‘snowballing’/‘pearl growing’ [31] have been used in searches for the effectiveness of complex interventions and in qualitative research. Including such approaches may add value, but their usefulness has not yet been evaluated for prognosis searches and may not be applicable to all cases. Further, the aim of the primary studies may not be the same of the systematic review, particularly where prognostic utility is a secondary outcome; it may thus not be immediately obvious that primary studies contain relevant information and a wider and more thorough assessment for study selection is likely to be required.

As a consequence of the difficulties in identifying relevant studies, there is variable inclusion of studies into systematic reviews or other evidence summaries. Added to this are varying approaches to analyses and dealing with heterogeneity, which has resulted in meta-analyses (where performed) obtaining different effect estimates and confidence intervals, using different types of outcome statistics and pooling studies with varying levels of heterogeneity. This has implications where a single systematic review, or even a sub-set of reviews, is being used to inform clinical opinion.

It is apparent that approaches to quality assessment have developed over time, with more recent systematic reviews [32, 33] using tools such as QUIPS. A degree of tailoring to the specific topic is likely to be appropriate, for example, if there are known confounders (in this area one of the main potential confounders was compliance). Only two of the eight systematic reviews used quality assessment to inform the discussion around their findings, which suggests that the importance of quality assessment is not being sufficiently recognised. Whilst this article explored how primary studies were quality assessed in the individual systematic reviews, the overall quality of the reviews was not formally assessed. There is currently no validated tool for assessing the quality of a systematic review of prognostic studies specifically; it is the intention of ROBIS [34], a new quality assessment tool under development, to be adaptable to systematic reviews of studies of different designs. An adapted GRADE framework [35] has also recently been proposed for use in conducting systematic review of prognostic factor research, which may improve the reporting and/or conduct of systematic reviews in this area.

Both quality assessment and interpretation of findings of prognostic studies are more complex where the identification of a prognostic factor is dependent on one or more diagnostic tests, with associated test accuracy issues. There is an additional layer of complexity where the potential prognostic factor is one of several, and is linked to several outcomes, as was the case in this example. As discussed in Dahlen et al. [36], it is not sufficient to demonstrate that there is a statistically significant association between a test result and a given outcome, as this may not be clinically useful.

Adjusted results show the incremental value of adding a (new) prognostic factor to existing ones for predicting an individual’s future risk [37]. The prognostic value is best examined on its continuous scale rather than dichotomising (e.g. actual blood pressure measurement rather than ‘high’ or ‘normal’ blood pressure), as this is more powerful and avoids arbitrary cut-off points. Therefore, where the prognostic question is complex and the reporting and quality of primary studies is poor, it is worth considering whether all primary studies need to be reviewed in detail, or whether it may be sufficient to examine those reporting adjusted results (from regression modelling). Studies presenting only unadjusted results may be of limited value and could be restricted to those deemed to be of higher quality (e.g. prospective study design), though reporting is likely to be an issue.

Some primary studies may report prognostic models, where the impact of adding the potential factor of interest to a model is explored, and an individual’s risk can be determined, as opposed to an average risk. However, different approaches to building models may hamper synthesis of results across (model based) studies, so including such studies in a systematic review is not without difficulties. It is unlikely that model-based studies will be available for all questions of interest; none were included in any of the reviews assessed in this article.

Where individual participant data (IPD) is available for analysis, this could negate some of the issues affecting the conclusions of such a review. IPD allows analyses based on the raw data from all studies, as opposed to aggregate (study) level data, and as such provides greater power to detect any prognostic effect. With available IPD, re-analysis could be performed to ensure that all studies provided the same effectiveness statistics. The impact of heterogeneity could be explored by analyses excluding subsets of patients, for example a particular trial using a different study design. Using IPD may also allow for the investigation of adjusted prognostic effects, which, as discussed, could provide a greater estimate of the true prognostic value of factors of interest. Using IPD data is however a resource-intensive and methodologically challenging process, and problems around availability bias, poor reporting and heterogeneity may not be overcome [38, 39].

We acknowledge that using our own systematic review as one of the example reviews may entail an inherent bias. This is a very recent review, and thus it may have been easier to incorporate more recent recommendations. The aim of this article was not to critique individual reviews but to identify common areas with potential for methodological development. Further, only one clinical area/question has been considered as an example in this article. It is hoped that the considerations outlined in Table 4 will add to the growing body of methodological recommendations in this field and aid those both using and undertaking systematic reviews of prognostic questions. This in turn may lead to better research recommendations and more robust primary studies. There are some similarities between our considerations and those identified in another clinical area (low back pain [4]), and this suggests generalizability to further clinical areas—indeed, similar methodological issues are currently being encountered in an ongoing systematic review [40] of prognostic factors and prognostic models for the recurrence of venous thromboembolism (VTE) following treatment for a first idiopathic VTE. There are also some more specific recommendations, e.g. around defining the prognostic factor, and approaches to analysis and study inclusion that have not previously been addressed.

Poorly conducted and reported primary prognostic research continues to hamper the potential of systematic reviews to inform on clinical questions. However, well-conducted reviews, which highlight uncertainty and heterogeneity in primary research, will be able to inform future primary studies. The utility of conducting further primary studies on individual prognostic factors should however also be considered; it may be more appropriate to focus on model-based studies, potentially using IPD data.

## Conclusions

Systematic reviews of prognostic factors are hampered by poor reporting of primary research [41], and the ability of systematic reviews of prognostic factors to inform on clinical questions is complicated by the utilisation of different methodological approaches.

Differences in approaches to study selection, synthesis and presentation of results may have the potential to influence the overall findings, which in turn may have implications for clinical decision-making, particularly where clinical opinion is being informed by a single systematic review.

Conclusions may be influenced by an overall effect size (and precision) where meta-analysis has been undertaken and by the interpretation of findings (e.g. extent of caveats applied). The potential for bias to be introduced into analyses through attempting to maximise data by making strong assumptions can also be significant. As with all evidence synthesis, inconsistency in the evidence must be accounted for in the careful interpretation of results. A hierarchy of evidence should be considered with adjusted results providing the greatest evidence for true prognostic value.

The findings from this article, based on a review of systematic reviews in one clinical area, have been used to generate a number of recommendations for those undertaking systematic reviews of prognostic research. These include a step-wise hierarchical approach to study selection and suggested approaches to searching, defining the prognostic factor, quality assessment and analysis, particularly with regard to consideration of heterogeneity.

This work adds to and extends the growing body of methodological evidence in this field and may ultimately help to inform the debate about what constitutes good systematic review practice for questions of prognostic utility.

## Electronic supplementary material

Additional file 1: **Sample search strategy.** This shows the search strategy for identifying (systematic) reviews in MEDLINE. (PDF 87 KB)

Additional file 2: **Systematic review selection process and main characteristics of systematic reviews.** This includes a flow diagram of the systematic review selection process and a table with the main characteristics of the included systematic reviews. (PDF 96 KB)

Additional file 3: **Number and overlap of included studies (search period up to 2006).** This shows the number and overlap of primary studies included in the systematic reviews, adjusted to the same search time period in order to make results comparable. (PDF 53 KB)

Additional file 4: **Proportion of studies included in reviews relating to different platelet function tests.** This shows the different proportions of primary studies investigating a particular platelet function test included in the respective reviews. (PDF 182 KB)

## Abbreviation

PFT: platelet function test.
