# Supplementary material for: Methodological issues and recommendations for systematic reviews of prognostic studies: an example from cardiovascular disease
Source: Syst Rev. 2014 Dec 3;3:140. doi: 10.1186/2046-4053-3-140 (PMC4265412; doi:10.1186/2046-4053-3-140)
Supplement: Supplementary file 1 — Additional file 1: Sample search strategy. This shows the search strategy for identifying (systematic) reviews in MEDLINE. (PDF 87 KB) [file 13643_2014_307_MOESM1_ESM.pdf]

Additional file 1

Search strategy

The following sources were searched using a combination of text words and MeSH terms, and a filter for identifying reviews: MEDLINE, MEDLINE In Process, EMBASE, Cochrane database of systematic reviews (including Database of Abstracts of Reviews of Effects (DARE) and HTA), Conference Proceedings Citation Index and Science Citation Index. Searches were undertaken from inception to April 2012 and updated in May 2014.

Example search strategy (MEDLINE)

1 ((ASA or aspirin or acetylsalicylic or anti platelet or antiplatelet or anti-platelet) adj2 (respons$ or non-respons$ or respond$ or non-respond$ or resistance or resist$)).mp. [mp=title, abstract, original title, name of substance word, subject heading word, keyword heading word, protocol supplementary concept word, rare disease supplementary concept word, unique identifier]

2 (platelet adj (response or respond$ or reactivity)).mp. [mp=title, abstract, original title, name of substance word, subject heading word, keyword heading word, protocol supplementary concept word, rare disease supplementary concept word, unique identifier]

3 1 or 2

4 exp Aspirin/

5 exp Drug Resistance/

6 4 and 5

7 3 or 6

8 (platelet function adj (analys$ or analyz$)).mp. [mp=title, abstract, original title, name of substance word, subject heading word, keyword heading word, protocol supplementary concept word, rare disease supplementary concept word, unique identifier]

9 (platelet function adj (assay$ or test$)).mp.

10 Platelet Function Tests/

11 PFA-100.mp.

12 PlateletWorks.mp.

13 Platelet Mapping.mp.

14 Impact Cone.mp.

15 platelet analyser$.mp.

16 platelet analyzer$.mp.

17 multiplate.mp.

18 aggregometry.mp.

19 LTA.mp.

20 AA-induced LTA.mp.

21 lumiaggregometry.mp.

22 WBA.mp.

23 ULTEGRA assay.mp.

24 Impact-R.mp.
25 TRAP-6.mp.
26 TEG.mp.
27 s-TEG.mp.
28 thromboelastometry.mp.
29 ROTEM.mp.
30 VerifyNow.mp.
31 Verify-Now.mp.
32 VN-RPFA.mp.
33 VASP.mp.
34 VASP-P.mp.
35 platelet reactivity index.mp.
36 vasodilator-stimulated phosphoprotein phosphorylation assay$.mp.
37 T-Guide.tw.
38 T Guide.ti,ab.
39 xylum clot signature analyser.mp.
40 xylum clot signature analyzer.mp.
41 ASA test$.mp.
42 ASA assay$.mp.
43 AA-induced LTA.mp.
44 exp Platelet Count/ or platelet counting.mp.
45 thrombelastography.mp. or Thrombelastography/
46 thrombotic status analyser$.mp.
47 thrombotic status analyzer$.mp.
48 or/8-47
49 exp Cardiovascular Diseases/
50 exp Cerebrovascular Disorders/
51 exp Diabetes Mellitus/
52 or/49-51
53 48 and 52
54 (predict$ or prognos$).mp. [mp=title, abstract, original title, name of substance word, subject heading word, keyword heading word, protocol supplementary concept word, rare disease supplementary concept word, unique identifier]
55 48 and 54
56 7 or 53
57 7 or 55
58 56 or 57
59 exp animals/ not humans/
60 58 not 59
61 limit 60 to (yr="2012 - Current" and "reviews (best balance of sensitivity and specificity)")
