# Supplementary material for: Methodological issues and recommendations for systematic reviews of prognostic studies: an example from cardiovascular disease
Source: Syst Rev. 2014 Dec 3;3:140. doi: 10.1186/2046-4053-3-140 (PMC4265412; doi:10.1186/2046-4053-3-140)
Supplement: Supplementary file 2 — Additional file 2: Systematic review selection process and main characteristics of systematic reviews. This includes a flow diagram of the systematic review selection process and a table with the main characteristics of the included systematic reviews. (PDF 96 KB) [file 13643_2014_307_MOESM2_ESM.pdf]

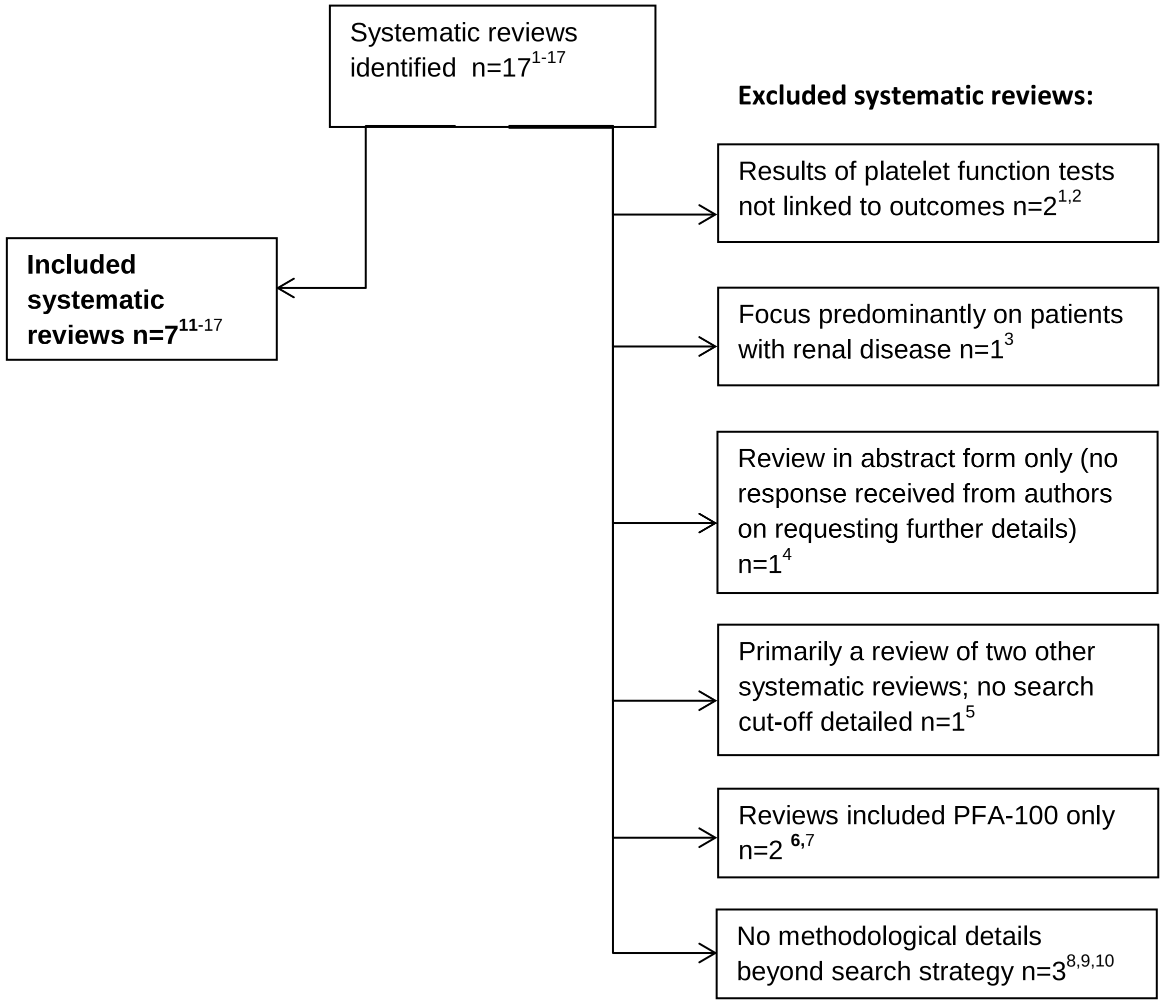

Figure: Systematic review selection process

Excluded
1. Verschuren JJ, Trompet S, Wessels JA, Guchelaar HJ, de Maat MP, Simoons ML, et al. **A systematic review on pharmacogenetics in cardiovascular disease: is it ready for clinical application?** *Eur Heart J* 2012; **33(2)**:165-175.

2. Dickinson KJ, Troxler M, Homer-Vanniasinkam S. **The surgical application of point-of-care haemostasis and platelet function testing.** *Br J Surg* 2008; **95(11)**:1317-1330.

3. El-Menyar A, Hussein H, Al SJ. **Coronary stent thrombosis in patients with chronic renal insufficiency.** *Angiology* 2010; **61(3):**297-303.

4. Velkovic J, Coulthard A. **The drugs don't work: Low response to antiplatelet agents in patients undergoing endovascular procedures**. *J Med Imaging Radiat Oncol* 2009; **53**:A275.

5. Musallam KM, Charafeddine K, Bitar A, Khoury M, Assaad S, Beresian J, et al. **Resistance to aspirin and clopidogrel therapy**. *Int J Lab Hematol* 2011; **33(1):**1-18.

6. Crescente M, Di CA, Iacoviello L, Vermylen J, Cerletti C, De GG. **Response variability to aspirin as assessed by the platelet function analyzer (PFA)-100. A systematic review**. *Thrombosis & Haemostasis* 2008; **99(1):**14-26.

7. Reny JL, de MP, Dauzat M, Fontana P. **Use of the PFA-100 closure time to predict cardiovascular events in aspirin-treated cardiovascular patients: a systematic review and meta-analysis**. *Journal of Thrombosis & Haemostasis* 2008; **6(3):**444-450.

8. Ferguson AD, Dokainish H, Lakkis N. **Aspirin and clopidogrel response variability: review of the published literature**. *Tex Heart Inst J* 2008; **35(3):**313-320.

9. Wong S, Appleberg M, Ward CM, Lewis DR. **Aspirin resistance in cardiovascular disease: a review**.*European Journal of Vascular & Endovascular Surgery* 2004; **27(5):**456-465.

10. Howard PA. **Aspirin resistance**. *Ann Pharmacother* 2002; **36(10):**1620-1624.

Included
11.Krasopoulos G, Brister SJ, Beattie WS, Buchanan MR. **Aspirin "resistance" and risk of cardiovascular morbidity: systematic review and meta-analysis.** *BMJ* 2008; **336(7637):**195-198.

12. Sofi F, Marcucci R, Gori AM, Abbate R, Gensini GF. **Residual platelet reactivity on aspirin therapy and recurrent cardiovascular events--a meta-analysis.** *Int J Cardiol* 2008; **128(2)**:166-171.

13 Canivano PL, Garcia YC. **[Resistance to aspirin: prevalence, mechanisms of action and association with thromboembolic events. A narrative review].** *Farm Hosp* 2010; **34(1):**32-43.

14. Pusch G, Feher G, Kotai K, Tibold A, Gasztonyi B, Feher A, et al. **Aspirin resistance: focus on clinical endpoints.** *J Cardiovasc Pharmacol* 2008; **52(6):**475-484.

15. Snoep JD, Hovens MM, Eikenboom JC, Van Der Bom JG, Huisman MV. **Association of laboratory-defined aspirin resistance with a higher risk of recurrent cardiovascular events: a systematic review and meta-analysis.** *Arch Intern Med* 2007; **167(15):**1593-1599.

16. Li J, Song M, Jian Z, Guo W, Chen G, Jiang G et al.: **Laboratory aspirin resistance and the risk of major adverse cardiovascular events in patients with coronary heart disease on confirmed aspirin adherence**. *J Atheroscler Thromb* 2014, **21**: 239-247.

17. Wisman PP, Roest M, Asselbergs FW, de Groot PG, Moll FL, van der GY et al.: **Platelet-reactivity tests identify patients at risk of secondary cardiovascular events: a systematic review and meta-analysis**. *J Thromb Haemost* 2014, **12**: 736-747.

Characteristics of included systematic reviews

Review	Research question	SR Methodology	Search date	Study eligibility criteria	Patients on aspirin mono or dual therapy (aspirin and clopidogrel) eligible	Outcomes	No. of included studies
Canivano Petrenas 2010 (SPANISH)	Prevalence, epidemiology, mechanism of action and clinical consequences of aspirin resistance.	Details of search strategy. Some detail on selection criteria. No further methodological details.	November 2008	-Patients over 18 treated with aspirin for the secondary prevention of cardiovascular or cerebrovascular events<br>-Definition of aspirin resistance<br>- Definition of methods for platelet function measurement	Not specified.	Cardiovascular and cerebrovascular events	16
Dretzke (in press)<br>HTA report	The prognostic and diagnostic utility of tests of platelet function for the detection of "aspirin resistance" in patients with established cardiovascular or cerebrovascular disease	Details of search strategy, study selection, quality assessment, data extraction and methods of analysis.	April 2012	-Any prospective primary studies, or systematic reviews of such studies, assessing platelet function test(s) in relation to clinical outcomes.<br>-Patients aged ≥18 years on aspirin (as monotherapy or in combination with other antiplatelet agents), with established cardiovascular or cerebrovascular disease, or diabetes.<br>-Either aspirin specific platelet function test or global platelet function test where patients are receiving aspirin as the only antiplatelet therapy; or aspirin specific platelet function test where patients are on dual/triple antiplatelet therapy (with aspirin as one of the agents).<br>-Reported outcomes had to occur after the undertaking of a platelet function test and the post-test follow-up period had to be seven days or longer.	Both	Clinical outcomes, such as vascular events, mortality, mortality due to vascular events and composite outcomes containing these.	102 (58 aspirin monotherapy)

Review	Research question	SR Methodology	Search date	Study eligibility criteria	Patients on aspirin mono or dual therapy (aspirin and clopidogrel) eligible	Outcomes	No. of included studies
Li 2013	The relationship between laboratory aspirin resistance and major adverse cardiovascular events in coronary heart disease patients with verified aspirin compliance	Details of search strategy, study selection, quality assessment, data extraction and methods of analysis.	April 2013	-Prospective study design<br>-Patients with CHD<br>-Aspirin treatment for the secondary prevention of events<br>-Definition of laboratory aspirin resistance clearly defined<br>-Patients classified into aspirin resistant/sensitive before observation of adverse events<br>-Compliance with aspirin confirmed<br>-Measurement of adverse events (and data reported) in both groups	Not specified.	Major adverse cardiovascular events	9
Krasopoulos 2008	Relationship between aspirin "resistance" and clinical outcomes in patients with cardiovascular disease.	Details of search strategy, study selection, quality assessment and methods of analysis.	Unclear ("to present")	-Participants were receiving aspirin therapy as an antithrombotic<br>-Participants were classified prospectively as aspirin sensitive or resistant<br>-There was *"adequate allocation concealment such that investigators were blinded to the patients' aspirin sensitive and aspirin resistant status"*<br>-A measure of prospective clinical outcome was used in both groups<br>-Patients receiving other antiplatelet treatment were also included	Mono or dual.	Clinical outcomes	20
Pusch 2008	Prevalence of aspirin resistance and its association with clinical outcome; treatment approaches.	Details of search strategy. Some detail on selection criteria. No further methodological details.	March 2008	-Articles that addressed aspirin resistance and nonresponsiveness and its diagnosis, clinical outcome and treatment	Not specified	Clinical endpoints	35

Review	Research question	SR Methodology	Search date	Study eligibility criteria	Patients on aspirin mono or dual therapy (aspirin and clopidogrel) eligible	Outcomes	No. of included studies
Snoep 2007	Relationship of laboratory aspirin resistance to risk of cardiovascular recurrent events.	Details of search strategy, study selection, quality assessment and methods of analysis.	October 2006	-Patients with established coronary artery, cerebrovascular or peripheral artery disease<br>-Patients treated with aspirin for secondary prevention of cardiovascular events<br>-Clear description of method used to establish effect of aspirin on platelet reactivity<br>-Reporting of data on recurrence rates of fatal and non-fatal MI, stroke or other cardiovascular outcomes as predefined by investigators	Not specified.	Fatal and non-fatal MI, stroke or other cardiovascular outcomes as predefined by investigators	16
Sofi 2008	Residual platelet reactivity in coronary heart disease patients in relation to the occurrence of adverse coronary events during follow-up.	Details of search strategy, study selection, data extraction and methods of analysis.	May 2007	-Prospective study design<br>- Coronary heart disease patients<br>-Patients on aspirin therapy for secondary prevention of cardiovascular events<br>-Clear description of residual platelet reactivity<br>-Clear description of methods used to identify residual platelet reactivity<br>-Relative risk, hazard ratio or odds ratio with 95% CIs (or data to calculate them)	Not specified.	Major adverse cardiovascular events	11

Review	Research question	SR Methodology	Search date	Study eligibility criteria	Patients on aspirin mono or dual therapy (aspirin and clopidogrel) eligible	Outcomes	No. of included studies
Wisman 2014	To assess which platelet function tests can reliably identify patients at risk of developing secondary cardiovascular events whilst receiving aspirin and/or clopidogrel treatment.	Details of search strategy, study selection, data extraction, quality assessment and methods of analysis.	October 2013	-Prospective study design<br>-Clear description of aspirin and/or clopidogrel regimen<br>-PFT with clear description of high platelet reactivity<br>-PFTs performed at baseline<br>-Clearly defined cardiovascular endpoints	Mono and dual	Primary: composite cardiovascular endpoint (cardiovascular death, MI, stent thrombosis, stroke, acute limb ischaemia, peripheral vascularisation, acute peripheral occlusion)<br><br>Secondary: occurrence of stent thrombosis, surrogate markers for cardiovascular damage	55 (27 aspirin monotherapy)
