# Supplementary material for: Methodological issues and recommendations for systematic reviews of prognostic studies: an example from cardiovascular disease
Source: Syst Rev. 2014 Dec 3;3:140. doi: 10.1186/2046-4053-3-140 (PMC4265412; doi:10.1186/2046-4053-3-140)
Supplement: Supplementary file 3 — Additional file 3: Number and overlap of included studies (search period up to 2006). This shows the number and overlap of primary studies included in the systematic reviews, adjusted to the same search time period in order to make results comparable. (PDF 53 KB) [file 13643_2014_307_MOESM3_ESM.pdf]

Additional file 3

Number and overlap of included studies (search period up to 2006)

	Canivano-Petrenas 2010[1]	HTA report (in press)[2]	Krasopoulos 2008[3]	Li 2013[4]	Pusch 2008[5]	Sofi 2008[6]	Snoep 2007[7]	Wisman 2014[8]
Sorensen 1983		■						
Grotemeyer 1993	■	■	■		■		■	■
Mueller 1997	■	■	■		■		■	■
Buchanan 2000	■	■	■	■	■		■	■
Eikelboom 2002	■	■			■		■	
Ziegler 2002		■					■	■
Andersen 2003	■		■		■	■	■	
Christiaens 2003	■	■						
Grundmann 2003	■		■		■		■	
Gum 2003	■	■	■		■	■	■	■
Gurbel 2003			■					
Bruno 2004		■			■			
Chen 2004	■		■		■		■	■
Cotter 2004		■	■		■		■	■
Faraday 2004			■		■			■
Payne 2004		■						
Sambola 2004		■						
Borna 2005			■		■			
Chen 2005	■		■					
Cheng 2005							■	
Hobikoglu 2005			■		■			
Morawski 2005		■						
McCabe 2005			■					
Pamukcu 2005					■			
Tantry 2005					■			
Yilmaz 2005	■		■		■		■	
Zhang 2005			■		■			
Berroushot 2006	■		■		■			
Cornelissen 2006					■			
Cuisset 2006		■				■		
Fuchs 2006		■				■		
Gianetti 2006		■				■		■
Lev 2006					■		■	
Marcucci 2006		■			■	■		■
Ohmori 2006		■			■			■
Pamukcu 2006	■	■		■	■	■	■	■
Poston 2006	■		■		■		■	
Stejskal 2006	■	■	■		■	■	■	
