# Supplementary material for: Methodological issues and recommendations for systematic reviews of prognostic studies: an example from cardiovascular disease
Source: Syst Rev. 2014 Dec 3;3:140. doi: 10.1186/2046-4053-3-140 (PMC4265412; doi:10.1186/2046-4053-3-140)
Supplement: Supplementary file 4 — Additional file 4: Proportion of studies included in reviews relating to different platelet function tests. This shows the different proportions of primary studies investigating a particular platelet function test included in the respective reviews. (PDF 182 KB) [file 13643_2014_307_MOESM4_ESM.pdf]

Proportion of studies included in reviews relating to different platelet function tests

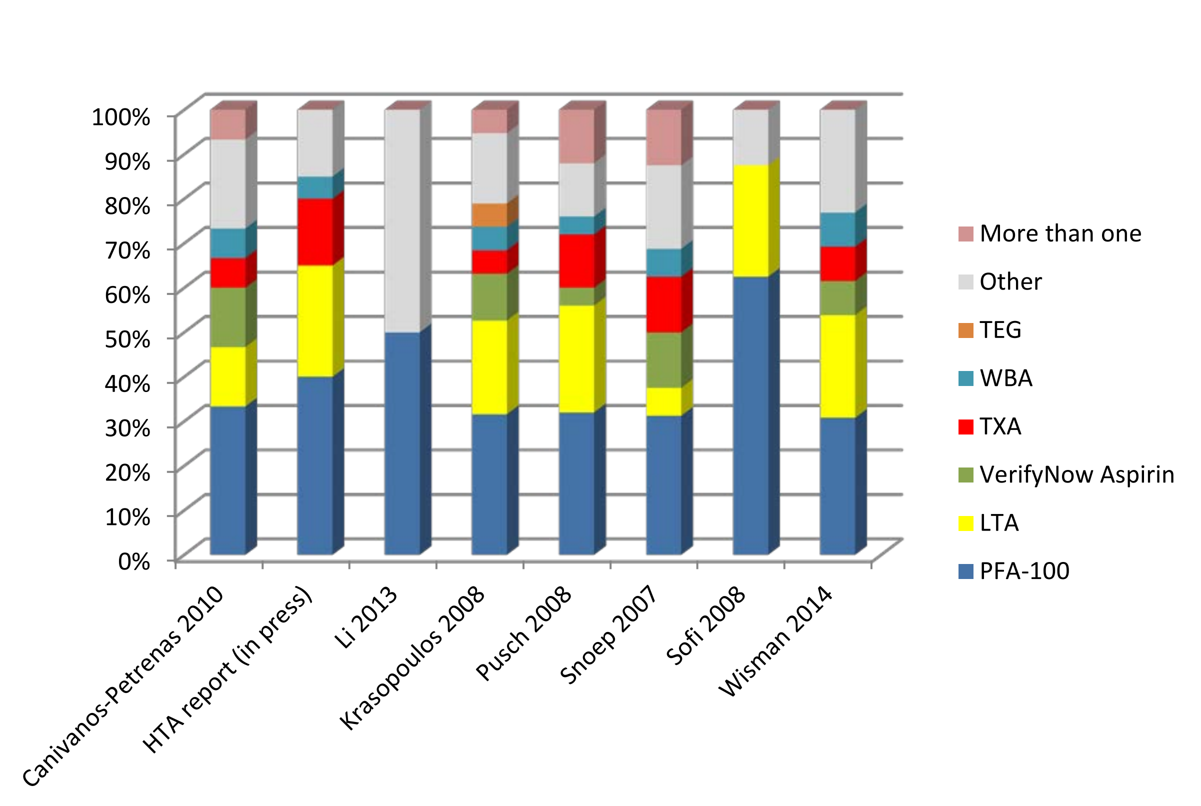

LTA=Light transmission aggregometry; PFA=platelet function analyser; TEG=thromboelastography; TXA=thromboxane; WBA=whole blood aggregometry. Varying number of studies in reviews (2 – 25).

"Other" includes: Apact II cationic propyl gallate platelet aggregometry, Surgicutt II bleeding time, Impact-R (Cone and Platelet Analyzer) and flow cytometry.
